# Impact of Gastroenterology Consultation on the Clinical Outcomes of Patients Admitted With Hepatic Encephalopathy

**DOI:** 10.7759/cureus.41610

**Published:** 2023-07-09

**Authors:** Nirjhar Dutta, Mandip KC, Qi Wang, Nicholas Lim

**Affiliations:** 1 Division of Hospital Medicine, University of Minnesota, Minneapolis, USA; 2 Division of Gastroenterology, Hepatology and Nutrition, University of Minnesota, Minneapolis, USA; 3 Clinical and Translational Science Institute, University of Minnesota, Minneapolis, USA

**Keywords:** 90-day mortality, 30-day readmission, gastroenterology consultation, hospital length of stay, hepatic encephalopathy

## Abstract

Introduction

Hepatic encephalopathy (HE) is a common complication of cirrhosis and a common reason for hospital admission. We aimed to determine whether expert consultation from gastroenterology (GI) leads to better clinical outcomes for inpatients with HE.

Methods

A retrospective review was performed of all adult patients (age ≥ 18) admitted with HE to a tertiary care hospital between January 2013 and April 2018. Patients who received a GI consult were compared to patients who did not receive a GI consult (No consult group). The primary outcome was hospital length of stay (LOS); secondary outcomes were rates of 30-day hospital readmission and 90-day mortality. Multivariate analysis was conducted to adjust for known confounders.

Results

Four hundred and twenty-five patients (814 encounters) were included in the study; of these, 236 patients had received a GI consultation for HE. Patients in the GI consult group were younger (mean age 55 vs 58 years, p= 0.02) and had higher Model For End-Stage Liver Disease-sodium (MELD-Na) score (mean MELD-Na 23.5 vs 17.5, p<0.01) compared to patients who did not receive GI consultation. The precipitants of HE were significantly different between the groups: there was more spontaneous bacterial peritonitis (SBP) and GI bleeding (GIB) in the GI consult group and more lactulose non-adherence in the no consult group. There was no difference in the etiology of liver disease between the two groups. Median LOS for the GI consult group was six days vs three days in the no consult group (p<0.01); the incidence rate ratio was 1.79 (95%CI 1.59-2.02, p<0.01) on multivariate analysis. There was no difference in 30-day readmission or 90-day mortality between the two groups.

Conclusion

GI consultation for patients with HE admitted to a hospital medicine service may be associated with longer LOS. In selected patients admitted with HE, GI consultation may not be necessary to achieve good clinical outcomes.

## Introduction

Hepatic encephalopathy (HE) is a common complication of cirrhosis and a common reason for hospital admission. HE is characterized by neuropsychological abnormalities and may present in a variety of manners ranging from subclinical changes to coma [1]. HE is also associated with higher rates of healthcare utilization and higher healthcare costs while admissions for HE are increasing [2,3]. Due to the high risk of clinical deterioration and complex management of patients with decompensated cirrhosis, gastroenterology (GI) consultation is often obtained for inpatients with HE. One large database study of United States Medicare enrollees with cirrhosis and prescription drug coverage showed that GI consultation was associated with lower rates of mortality and 30-day readmissions [4]. However, no study to date has specifically looked at the impact of GI consultation on patients admitted to the hospital with HE. The aim of this study was therefore to determine whether GI consultation leads to improved clinical outcomes of patients admitted with HE.

## Materials and methods

We conducted a retrospective review of electronic health records (EHR) for patients admitted with HE between January 2013 and April 2018 at a Midwestern academic health center. The study was carried out in M Health Fairview University of Minnesota Medical Center, Minneapolis, Minnesota, United States. The study was approved by the University of Minnesota Institutional Review Board (approval no: STUDY00001470) and the requirement for informed consent was waived due to the use of de-identified routine clinical data. Patients who opted out of having their clinical data used for research purposes were excluded.

Patients were initially identified using the International Classification of Diseases, Ninth Revision (ICD-9) or tenth revision (ICD-10) admission or discharge codes for cirrhosis, HE, and/or altered mental status, and then validated by manual chart review by a board-certified internal medicine physician (hospitalist) to meet American Association for the Study of Liver Diseases (AASLD) practice guideline criteria for HE [5]. Adult patients (age ≥ 18) with cirrhosis who met the criteria for hepatic encephalopathy were included. Patients with a liver transplant (LT), hospice/comfort care patients, patients admitted for another acute neurological condition (e.g. stroke, intoxication, meningitis), or admitted to the intensive care unit or a non-medicine service were excluded. 

Demographics (age, gender, and ethnicity), hospitalization data (admission and discharge dates, hospital readmission data, hospital admission ward), laboratory values, medications, and comorbidity conditions were obtained from the EHR. Etiology of liver disease and precipitating factors for HE was obtained by manual chart review. Patient demographics, clinical characteristics, and outcomes were summarized using descriptive statistics. Patients who received a GI consult were compared to patients who did not receive a GI consult (no consult group). The primary outcome was hospital length of stay (LOS), while secondary outcomes were rates of 30-day hospital readmission and 90-day mortality. 

Data analysis

The GI consult group and the no consult group were compared using two-sample t-test (or nonparametric Wilcoxon test with skewed distribution) for continuous variables and Chi-square test (or Fisher’s exact test with sparse data) for categorical variables. Multivariate analysis was conducted to adjust for covariates including age at first HE episode, gender, race, etiology of liver disease, rifaximin use, and precipitant for HE. These covariates were selected based on scientific rationale as clinical factors known to affect clinical outcomes in patients with HE. The Model For End-Stage Liver Disease-sodium (MELD-Na) score was not included in the multivariate analysis model due to the high percentage of missing data; sensitivity analysis with the MELD-Na score in the model did not statistically change the estimates. Binary outcomes were modeled by logistic regression. LOS was modeled by negative binomial regression. All analyses were conducted using SAS version 9.4 (SAS Institute Inc., Cary, North Carolina, United States).

## Results

Baseline characteristics

A total of 425 patients (814 encounters) were included in the study; of these, 236 patients had received a GI consultation for HE. Patients in the GI consult group were younger (mean age 55 vs 58 years, p= 0.02) and had higher MELD-Na score (mean MELD-Na 23.5 vs 17.5, p<0.01) compared to patients who did not receive GI consultation (Table 1). 

**Table 1:** Baseline characteristics MELD-Na: Model For End-Stage Liver Disease-sodium

	No GI consult	GI consult	P- value
Number of patients	189	236	
Number of encounters	522	292	
Female, N (%)	81 (43%)	86 (36%)	0.15
Age, mean (SD)	58.0 (11.6)	55.5 (10.3)	<0.05
White, N (%)	144 (83%)	190 (87%)	0.28
MELD-Na, mean (SD)	17.5 (9.5)	23.5 (8.7)	<0.0001
Rifaximin use, N (%)	450 (86%)	263 (90%)	0.11
Etiology of Cirrhosis, N (%)			0.10
Alcohol	70 (39%)	111 (49%)	
Hepatitis C virus	28 (15%)	26 (12%)	
Alcohol and hepatitis C Virus	24 (13%)	19 (8%)	
Other	59 (33%)	70 (31%)	
Precipitant of Hepatic Encephalopathy			<0.0001
Medical non-adherence	204 (39%)	73 (25%)	
Spontaneous bacterial peritonitis	17 (3%)	30 (10%)	
Gastrointestinal bleeding	13 (2%)	22 (8%)	
Urinary tract infection	52 (10%)	24 (8%)	
Other	136 (26%)	83 (28%)	
Unknown	100 (19%)	60 (21%)	

Lactulose non-adherence, spontaneous bacterial peritonitis, gastrointestinal bleeding, and urinary tract infections were the most common precipitants of HE. Other precipitants of HE were the following: transjugular intrahepatic portosystemic shunt procedure, opioid medication use, constipation, dehydration, infection (pneumonia, cholangitis, bacteremia), and electrolyte abnormalities (hyponatremia, hypokalemia). The precipitants of HE were significantly different between the groups; there was more spontaneous bacterial peritonitis (SBP) and GI bleeding (GIB) in the GI consult group. Lactulose non-adherence was the most common precipitant in the no consult group. No precipitant of HE was identified in about 20% of patients in both groups. There was no difference in the etiology of liver disease between the two groups. 

Outcomes

The primary outcome of median LOS for the GI consult group was six days compared to three days in the no consult group (p<0.01; incidence rate ratio 1.79; 95%CI 1.59-2.02, p<0.01 on multivariate analysis) (Table 2 and Table 3). 

**Table 2:** Primary and secondary outcomes * Incident rate ratio, negative binomial regression ** Odds Ratio, logistic regression Adjustments were made for age, sex, race, rifaximin use, hepatic encephalopathy precipitant, and etiology of cirrhosis

Outcome	GI consult group	No consult group	GI consult group vs. No consult group, 95% CI	P-value
Hospital length of stay (median, range)	6 days (3-10)	3 days (2-5)	1.79* (1.59-2.02)	<0.0001
30-day hospital readmission	52%	39%	1.36** (0.88-2.11)	0.17
90-day mortality post hospital discharge	38%	31%	1.22** (0.77-1.93)	0.40

**Table 3:** Multivariate analysis of hospital length of stay * Negative binomial regression on encounter level data. HE: hepatic encephalopathy; GIB: gastrointestinal bleeding; Lac-NA: Lactulose non-adherence; other: other causes; SBP: spontaneous bacterial peritonitis; UNK: unknown; UTI: urinary tract infection; ETOH: alcohol; HEPC: Hepatitis C virus

		Incident Rate Ratio (95% confidence interval)*	P- value
Gastroenterology Consultation	Yes vs. No	1.79 (1.59-2.02)	<0.0001
Age	per one-year increase	1.00 (0.99-1.00)	0.09
Sex	Female vs Male	1.13 (0.99-1.29)	0.07
Race	Nonwhite vs White	1.05 (0.90-1.22)	0.55
Etiology of cirrhosis	ETOH+HEPC vs ETOH only	0.85 (0.69-1.04)	0.11
	HEPC only vs ETOH only	0.88 (0.72-1.07)	0.20
	Other vs ETOH only	0.96 (0.84-1.11)	0.61
HE precipitant	GIB vs Lac-NA	1.58 (1.20-2.10)	<0.05
	Other vs Lac-NA	1.40 (1.20-1.63)	<0.0001
	SBP vs Lac-NA	1.78 (1.39-2.27)	<0.0001
	UNK vs Lac-NA	1.33 (1.12-1.57)	<0.05
	UTI vs Lac-NA	1.39 (1.11-1.74)	<0.05
Rifaximin use	Yes vs. No	1.14 (0.95-1.37)	0.15

There was no statistically significant difference in the 30-day readmission rate; 52% of patients in the GI consult group vs 39% of patients in the no consult group were readmitted within 30 days (OR 1.36; 95%CI 0.88-2.11; p= 0.11 on multivariate analysis) (Table 4). 

**Table 4:** Multivariate analysis of 30-day hospital re-admission *Logistic regression on patient-level data HE: hepatic encephalopathy; GIB: gastrointestinal bleeding; Lac-NA: lactulose non-adherence; other: other causes; SBP: spontaneous bacterial peritonitis; UNK: unknown; UTI: urinary tract infection; ETOH: alcohol; HEPC: hepatitis C virus

		Odds Ratio (95% confidence interval)*	P-value
Gastroenterology consultation	Yes vs. No	1.36 (0.88-2.11)	0.17
Age	per one year increase	0.99 (0.96-1.00)	<0.05
Sex	Female vs Male	0.67 (0.42-1.10)	0.09
Race	Nonwhite vs White	1.01 (0.54-1.89)	0.99
Etiology of cirrhosis	ETOH+HEPC vs ETOH only	0.88 (0.41-1.89)	0.40
	HEPC only vs ETOH only	1.17 (0.59-2.34)	0.84
	Other vs ETOH only	1.49 (0.90-2.46)	0.14
HE precipitant	GIB vs Lac-NA	0.96 (0.36-2.57)	0.51
	Other vs Lac-NA	1.11 (0.62-1.98)	0.59
	SBP vs Lac-NA	1.30 (0.57-2.94)	0.90
	UNK vs Lac-NA	1.14 (0.60-2.17)	0.72
	UTI vs Lac-NA	2.38 (1.08-5.27)	<0.05
Rifaximin use	Yes vs. No	2.64 (1.39-5.01)	<0.05

There was no statistically significant difference in the 90-day mortality after hospital discharge between the groups (38% in the GI consult group vs. 31% in the no consult group (OR 1.22; 95%CI 0.77-1.93; p= 0.40) (Table 5). 

**Table 5:** Multivariate analysis of post-discharge 90-day mortality *Logistic regression on patient-level data HE: hepatic encephalopathy; GIB: gastrointestinal bleeding; Lac-NA: lactulose non-adherence; Other: other causes; SBP: spontaneous bacterial peritonitis; UNK: unknown; UTI: urinary tract infection; ETOH: alcohol; HEPC: hepatitis C virus

		Odds Ratio, (95% confidence interval) *	P-value
Gastroenterology consultation	Yes vs. No	1.22 (0.77-1.93)	0.40
Age	per one year increase	1.02 (0.99-1.04)	0.19
Sex	Female vs Male	1.87 (1.16-3.03)	<0.05
Race	Nonwhite vs White	0.59 (0.29-1.18)	0.13
Etiology cirrhosis	ETOH+HEPC vs ETOH only	1.93 (0.90-4.16)	<0.05
	HEPC only vs ETOH only	0.81 (0.38-1.71)	0.31
	Other vs ETOH only	0.83 (0.49-1.40)	0.22
HE precipitant	GIB vs Lac-NA	1.24 (0.44-3.50)	0.99
	Other vs Lac-NA	1.08 (0.59-1.99)	0.56
	SBP vs Lac-NA	1.33 (0.57-3.13)	0.83
	UNK vs Lac-NA	1.10 (0.56-2.17)	0.66
	UTI vs Lac-NA	1.80 (0.82-3.98)	0.22
Rifaximin use	Yes vs. No	1.67 (0.86-3.25)	0.13

## Discussion

HE is a common reason for hospital admission in patients with cirrhosis and is associated with higher rates of healthcare utilization. In this single-center study, GI consultation was associated with longer LOS in patients admitted for HE. Furthermore, in these patients, rates of readmissions and 90-day mortality did not appear to be affected by GI consultation. 

Recent studies have shown that GI consultation leads to increased adherence to quality indicators for ascites and esophageal varices in patients with cirrhosis although the impact on major clinical outcomes is less clear [6,7]. One single-center study investigated the impact of mandatory GI consultation for patients admitted with decompensated cirrhosis and reported increased compliance with QIs but no difference in LOS, readmissions, or inpatient mortality [8]. Furthermore, a recent large database study showed that GI consultation was only associated with lower rates of 30-day readmissions in a Veterans Affairs cohort and that this was not observed in a validation cohort of privately insured patients [9]. Similar findings have been seen in other areas of medicine: clinical outcomes were similar in patients undergoing cardiac catheterization regardless of cardiology consultation, and adherence to guidelines was also similar between patients admitted to the hospital with *Staphylococcus aureus* infection who did and did not receive an infectious diseases consultation [10,11].

In this study, GI consultation was obtained more often in patients with higher disease severity, as shown by a higher mean MELD-Na score at presentation, with these patients likely to have a more aggressive treatment approach requiring a longer LOS. The higher rate of GIB and SBP in the GI consult group likely contributed to longer LOS for these reasons. Society practice guidelines for the treatment of SBP recommend antibiotics and intravenous albumin on days one and three; similarly, guidelines for GIB in patients with cirrhosis recommend the use of antibiotics and often an endoscopic procedure [12,13]. Moreover, patients in the non consult group had higher rates of lactulose non-compliance as the precipitant of HE, which would be expected to respond to empiric therapy faster and, therefore, be ready for discharge sooner. 

Our results show that for selected patients with HE (e.g. precipitated by lactulose non-adherence), GI consultation may not always be necessary to achieve comparable clinical outcomes. While these selected patients may not always need formal consultation, good communication between hospitalists and gastroenterologists remains essential as patients with cirrhosis remain at high risk for clinical deterioration and hospital readmissions. Although physicians may have mixed views and experiences of curbside consultation, it may be reasonable to consider in this select population of patients with HE [14-16]. 

The strength of this study is that the data were reviewed meticulously by a physician and provide a level of granularity that cannot be captured by large database studies. As a single-center study, our results may not be generalizable to all practice settings and the retrospective study design has its inherent flaws. Our cohort wasn’t large enough to assess the timing of the consult (e.g. early, within 24 hours of admission, versus late, >24 hours of admission) which could play an important role in patient outcomes. Other important subgroup analyses such as subgroups by etiology of cirrhosis or precipitant of HE also could not be performed due to our sample size. Larger cohort studies and randomized trials across multiple centers should be undertaken to assess the timing of consultation and the impact of HE precipitant in this complex patient population. 

## Conclusions

Patients admitted to the hospital with HE who receive a GI consultation may have a longer LOS. Hospitalists may be more likely to obtain GI consultation on patients with higher disease severity (e.g. higher MELD-Na score) or for certain diagnoses (e.g. SBP or GIB) which can impact LOS. In selected patients with lower disease severity, GI consultation may not be necessary to achieve good clinical outcomes. 
